# Case Report: Interventional therapy for coronary artery occlusion in a 6-year-old child with Kawasaki disease

**DOI:** 10.3389/fped.2022.1048178

**Published:** 2022-12-05

**Authors:** Lu Zhao, Li-ping Xie, Lan He, Xue-cun Liang, Chen Chu, Fang Liu

**Affiliations:** Department of Cardiology, Children’s Hospital of Fudan University, National Children’s Medical Center, Shanghai, China

**Keywords:** kawasaki disease, CTO - percutaneous coronary intervention, CTO (chronic total occlusion), cornary artery, children

## Abstract

A 6-year-old girl was diagnosed with Kawasaki disease and bilateral giant coronary artery aneurysms at four months old and was subsequently referred to our hospital due to chest pain and T wave changes on electrocardiography. After admission, stress myocardial perfusion imaging showed reversible ischemia in multiple areas of the left ventricle. Coronary angiography revealed complete proximal segment occlusion of the left circumflex artery (LCX). The occluded LCX was recanalized by a Gaia 3rd micro-wire successfully passing through the occluded section to the distal end of the LCX, followed by sequential balloon dilation and drug-coated balloon angioplasty. Coronary angiography immediately after post-dilation and one-year follow-up angiography showed that the structure and blood flow of LCX was good. Although percutaneous coronary intervention (PCI) in pediatric patients with Kawasaki disease is limited in practice, PCI remains one of the treatment options for selected patients.

## Introduction

Kawasaki disease (KD) is a self-limiting vasculitis predominantly occurring in children from 6 months to 5 years old. Coronary artery lesions (CALs) occur in 25% of untreated children and 5%–10% treated children (1, 2). It is reported that 30%–50% of coronary artery aneurysms (CAAs) regressed to normal within two years. However, giant aneurysms with a diameter of ≥8.0 mm rarely revert. Patients with giant aneurysms may develop coronary artery thrombosis even with antiplatelet and anticoagulant therapy. Chronic coronary artery stenosis and occlusion due to thrombus formation in the lumen and/or thickening of the intima results in myocardial ischemia, myocardial infarction, and even sudden death (3). Coronary artery bypass grafting (CABG) is the mainstay therapy for such patients due to the complexity and severity of coronary artery disease caused by KD. The experience of percutaneous coronary intervention (PCI) for children is quite limited, especially in children with chronic total occlusion (CTO) of the coronary artery. Here we report a 6-year-old girl who developed a CTO of her left circumflex artery (LCX) and was successfully recanalized by an antegrade technique followed by drug-coated balloon dilatation.

## Case presentation

A 6-year-old girl was referred to our hospital for chest pain with fatigue (CCS class I). She was diagnosed with KD accompanied by coronary lesions at four months of age at a local hospital. Bilateral CAAs further aggravated to 10 mm in the LAD and 8.2 mm in the RCA at week six after onset and then remained stable. She was given oral aspirin and warfarin, with an international normalized ratio maintained at approximately 2.0. However, no further examination was done to evaluate CALs and myocardial ischemia except for regular echocardiography and electrocardiography until 30 months after onset, when coronary computed tomography angiography (CTA) demonstrated a locally dilated left coronary artery with a thickened wall and calcification. The diameters of the left main coronary artery (LMCA), LAD, LCX, and RCA were 3.8, 6.3, 2.6, and 3.7 mm, respectively. As such, warfarin was discontinued while aspirin was commenced at 62.5 mg once daily. She was then followed up irregularly at the local hospital. Six years after onset, a week before admission to our hospital, the patient had chest pain, dyspnea without syncope, and profuse sweating, which resolved after 20 min. An electrocardiogram at the local hospital demonstrated T wave changes. She was referred to our hospital for further evaluation and treatment.

**Figure 1:**
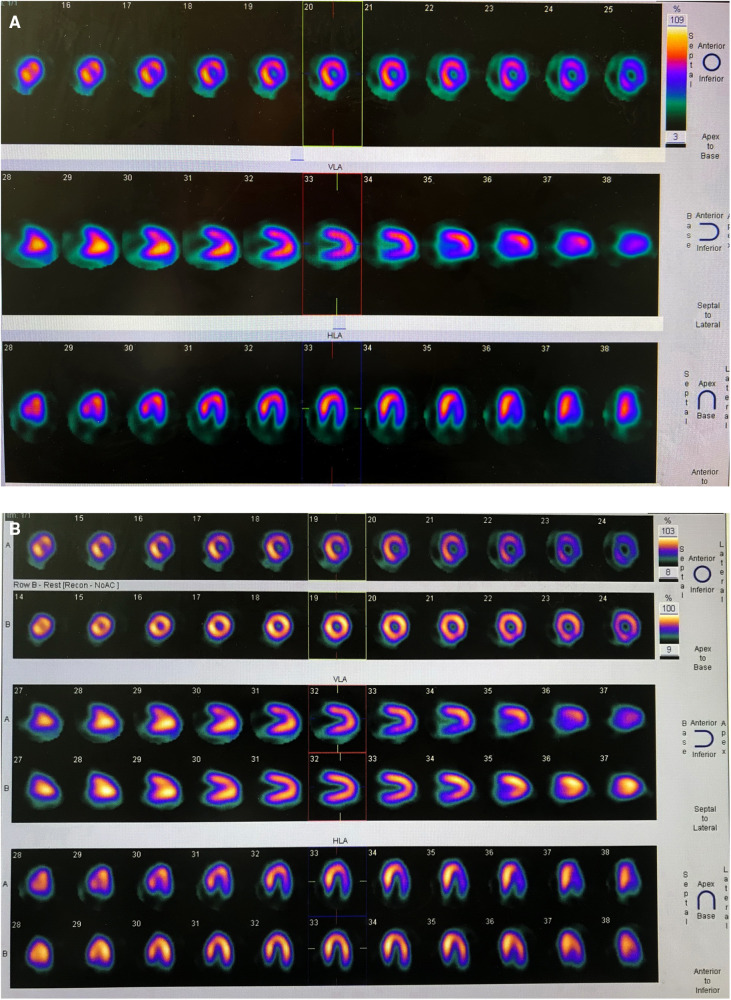
(**A**) ATP stressed myocardial perfusion imaging (MPI) demonstrated ischemia in the anterior wall near the apex, inferior wall, posterior wall, and lateral wall of the left ventricle. (**B**) Routine MPI showed uniform myocardial distribution.

**Figure 2:**
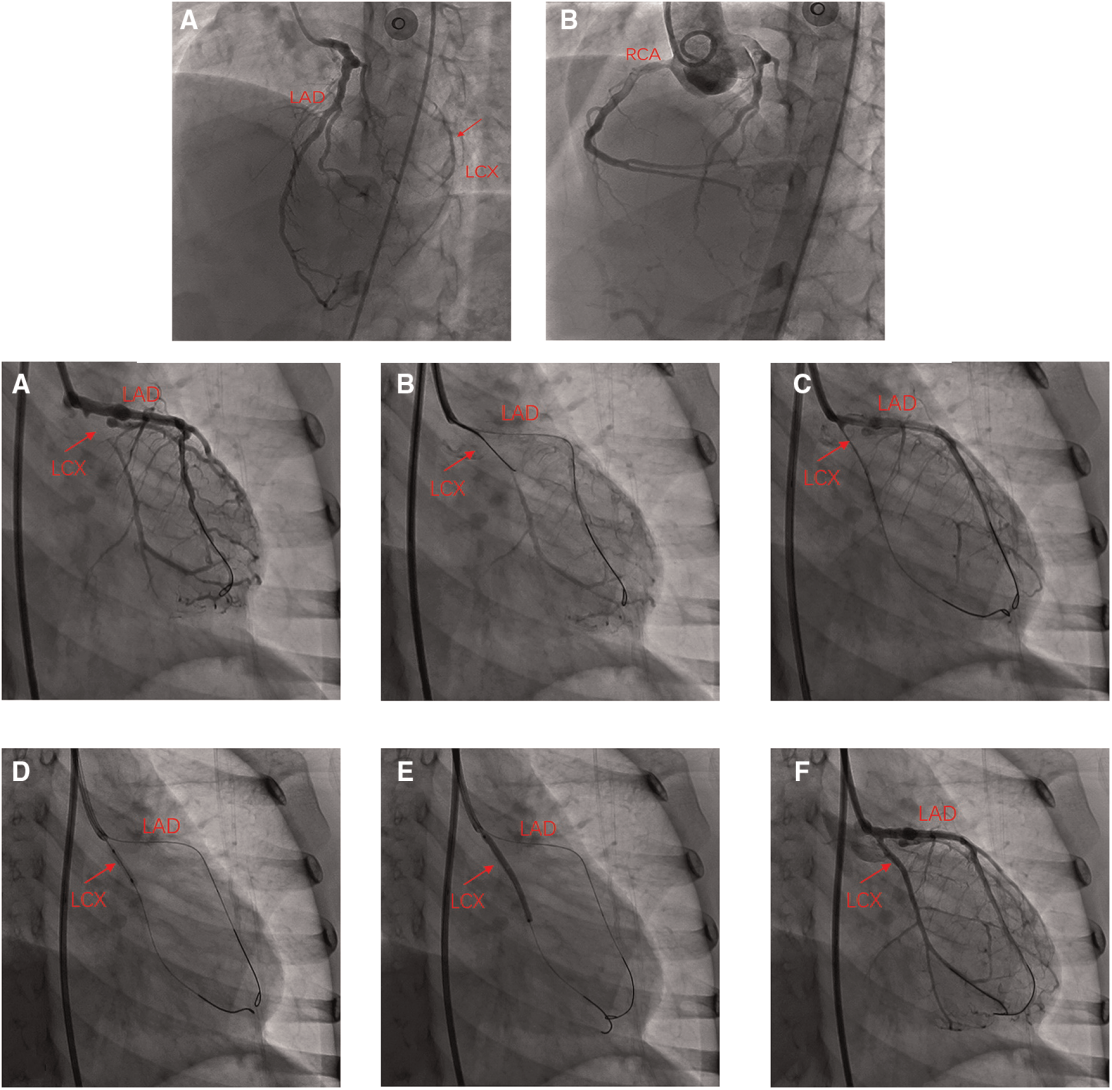
Angiography in left anterior oblique(LAO) 45° and cranial 25°: (A) total occlusion of the proximal LCX without calcification and collateral vessels from the LAD to distal LCX. (**B**) Internal diameter of the RCA was nearly average. (**A**) Angiography in right anterior oblique (RAO) 30°: LCX occluded at the proximal end, and distal blood supply has relied on collateral vessels; (**B,C**) Recanalization of occluded LCX using antegrade crossing technique; (**D**) Angiography after balloon angioplasty showed patent LCX; (**E**) DCB was attached to the LCX for 60 s; (**F**) Angiography of the LCX after DCB angioplasty.

**Figure 3:**
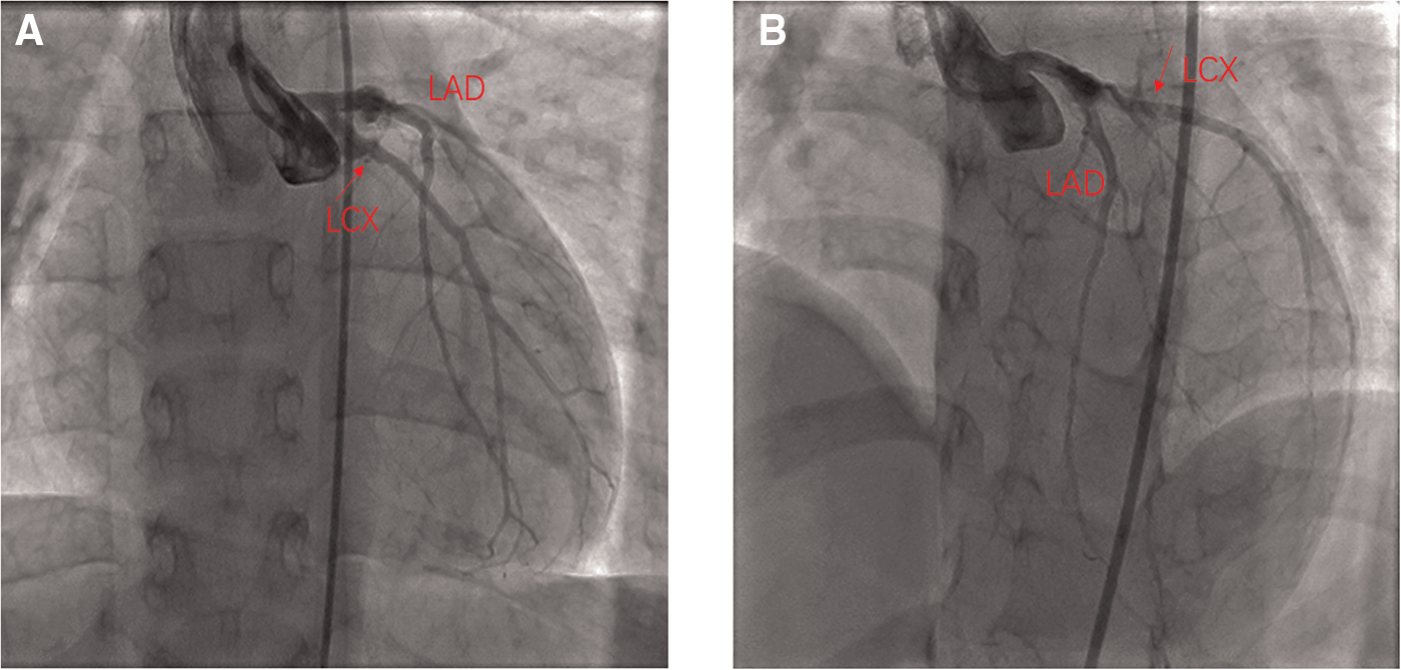
One year after the PCI angiography showed good flow in the LCX. (**A**) Angiography in right anterior oblique15° and Caudal 15°; (**B**) Angiography in left anterior oblique 45° and Cranial 25°.

On the day of admission, the patient was in good condition, and her body weight was 19.5 kg. Blood troponin I and N-terminal pro-B-type natriuretic peptide (NT-proBNP) were within normal limits. Electrocardiogram and treadmill test were normal while ATP stressed myocardial perfusion imaging (MPI) demonstrated reversible ischemia in the anterior wall near the apex and inferior, posterior, and lateral walls of the left ventricle (Figures 1A,B). Coronary angiography was performed and revealed a small coronary aneurysm (4.8 mm in diameter) in the LAD with severe calcification around the aneurysm wall, total occlusion of the proximal LCX without calcification, and collateral vessels from the LAD to the distal LCX (Figure 2A). The diameter of the RCA was near average with slight distortion in the main branch, suggesting a regression of previous aneurysms (Figure 2B). Following discussion amongst the cardiac multidisciplinary team (MDT), percutaneous revascularization for occlusive LCX was considered a suitable strategy for this patient. The procedure was performed under general anesthesia *via* the right femoral artery. She was heparinized (100 µ/kg), and the activated clotting time was monitored every hour, maintained at over 200 s. A 6-French JL3.5 guide catheter (Cordis) was advanced to the opening of the left coronary artery with great effort. A 0.014-in guidewire was introduced to the proximal segment of the LAD for support. A 2.6F (Corsair, Asahi) microcatheter was then introduced to the opening of the LCX (Figure 2A). Afterwards, attempts were made to pass Sion/Fielder XT-R/Fielder XT-A guide wires (Asahi) through the occlusion, but all attempts failed. Ultimately, a Gaia^3rd^ guidewire (Asahi) was successfully passed across the occlusion site (Figure 2B) to the distal LCX (Figure 2C). We gently performed pre-dilations of the occluded lesion with a 2.0 mm × 12 mm Emerge™ PTCA balloon (Boston Scientific) (Figure 2D) and further dilations with a 2.5 mm × 30 mm paclitaxel-coated balloon (Bingo, Yinyi Biological) at 7 atm for 60 s (Figure 2E). The final coronary angiography showed that the occluded segment of the LCX was successfully recanalized, with the contrast agent evenly filling and no arterial dissection detected (Figure 2F). The procedure concluded free of complications. After the procedure, the child continued to be administered aspirin 100 mg and clopidogrel 20 mg per day. Postoperative troponin I remained normal, and color doppler ultrasound showed good blood flow in the LCX. She was discharged from the hospital five days after the procedure. At one year of follow-up, the patient had no symptoms with good exercise tolerance. Her electrocardiogram, treadmill test and ATP stressed MPI were normal. Coronary artery angiography demonstrated good blood flow in the LCX (Figures 3A,B) and the complete blood count as within normal limits. The timeline of therapy for this patient is listed in (Table 1).

**Table 1:** Timeline of diagnosis and therapy of the patient.

Time	Event	Management
Local hospital		
age:4 months	Kawasaki disease was diagnosed	2 g/kg IVIG and 30–50 mg/kg aspirin were administrated
age:5 months	CAA was found by echocardiography, and the size of the CAA was stable.	Warfarin and 5 mg/kg aspirin were administrated in the local hospital. INR index was maintained at 2.0
age:3 years	Coronary artery CT scan showed calcification lesions in LCA. The size of CAA was decreased.	Warfarin was withdrawn. Oral aspirin was continued.
age:6 years	CT scan showed the calcification size in LCA increased, and LCX seems slender. The patient had a complaint of chest tightness and shortness.	Oral aspirin was continued.
Our hospital		
day 1–3	ECG monitored. Laboratory tests and imaging tests were performed.	Aspirin and beta-blocker were administrated.
day 4	Coronary artery angiography and PCI were performed.	
day 5	The result of ECG and blood serum cardiac troponin I were normal.	Oral aspirin, clopidogrel, and beta-blocker were continued.
day 9	Hospital discharge	
One year after PCI	ECG was normal. Stress MPI was normal.CA angiography showed LCX had a good blood flow.	Aspirin, clopidogrel, and beta-blocker were continued.

## Discussion

PCI is an important treatment method for acute coronary syndrome and chronic occlusive coronary artery disease in adults. However, the experience of PCI in KD children with CALs is minimal and reports are predominantly from Japan. The current PCI methods for KD specifically include balloon angioplasty, stent implantation, and intravascular rotational ablation (4). According to the JCS and AHA guidelines for KD, the indications for PCI in KD patients include the evidence of myocardial ischemia or inducible myocardial ischemia with coronary stenosis greater than 75%. CABG may be more beneficial for younger children or patients with the multiple-vessel disease (1, 2). In our case, ATP stressed MPI demonstrated reversible ischemia in multiple areas of the left ventricle, and coronary angiography showed complete occlusion of the proximal LCX. Therefore, indications for coronary intervention for this patient were absolute. Although the child had complex bifurcation lesions involving the LAD and LCX, the LAD was not indicated for coronary intervention. There was no significant reduction of blood flow in LAD, and the left ventricular function was as expected. Considering the focal lesion and the absence of calcification in the LCX, percutaneous revascularization for total occlusive LCX may be the first consideration. However, if the lesions in the LCX and LAD progress in the future, the procedure of CABG should be considered.

Reports on PCI in children with KD complicated with CTO of coronary arteries are rare. To our knowledge, there are only four reports in the literature. The recanalization method of occluded coronary arteries in children is the same as in adults, mainly relying on forward or reverse dissection reentry or forward true cavity to true cavity pathfinding technology. The youngest KD patient with total occlusion of coronary arteries undergoing PCI was reported to be six years old. After recanalization of the occluded coronary arteries was achieved, two patients underwent stent implantation, and the other two patients underwent balloon angioplasty. The longest follow-up period was 6 months. The short-term clinical effects were promising, but the long-term results still need follow-up (5–8). Our patient underwent paclitaxel-coated balloon angioplasty after recanalizing the LCX using the antegrade crossing technique. Her immediate and short-term postoperative outcome was also excellent. Drug-coated balloons (DCB) are a novel therapeutic strategy for coronary artery disease based on the rapid release of antiproliferative drugs into the local vessel wall during balloon inflation to inhibit intimal hyperplasia. In theory, DCB can effectively reduce the re-intervention rate of coronary lesions compared with angioplasty only. Recently, several large and adequately designed trials for the treatment of coronary small-vessel disease have confirmed the efficiency of the drug-coated balloon (9). However, the use of DCB in children is limited, especially in Kawasaki disease coronary cases. In 2021, Xu et al. reported the first successful percutaneous revascularization using a paclitaxel-coated balloon for coronary sequelae of KD in the pediatric population (8). In 2022, Zheng et al. reported another case about an 18-year-old girl who had severe stenosis in the proximal RCA sequelae with KD was treated with DCB and had an excellent result at one-year follow-up (10). Previously, limited case reports about paclitaxel-coated balloon treatment for renal artery, hepatic vein, and pulmonary artery stenosis in children had been published (11–13). To date, no severe adverse effects of the paclitaxel-coated balloon have been found in children. In our case, at one year after PCI, the clinical outcome of PCI and the blood tests were favorable and the patient had no obvious symptoms of immunosuppression. Nevertheless, longer follow-up on our patients and more studies on this topic is needed.

Stent implantation in patients with coronary arterial lesions secondary to KD should be considered carefully and be avoided or delayed as much as possible because coronary artery stenting in this population had some long-term adverse effects, including complete occlusion, restenosis, and stent migration. In 2020, Tsuda reviewed 33 patients who underwent stent implantations and reported that late-period adverse effects were found in 19 (68%) of 28 vessels with follow-up angiograms. The rate of being free of adverse effects at three years after the procedure was only 25% (14).

To date, few publications have compared the outcomes between CABG and PCI in KD children. Data is mainly derived from retrospective studies. Detailed comparisons between PCI and CABG were summarized in Table 2. Most studies affirm that KD patients undergoing PCI had a higher rate of re-intervention than those undergoing CABG (15, 16). However, PCI has the absolute advantages of being minimally invasive with a shorter recovery time for children. Therefore, PCI in selected KD patients with CALs can improve the prognosis and benefit these patients.

**Table 2:** PCI vs. CABG in CA lesions with KD.

	Advantages	Disadvantages
PCI	(1) Being minimally invasive with a shorter recovery time (2) May lead to clinical improvement and delay the need for surgical intervention.	(1) Long-term outcome is uncertain (2) May be have a higher rate of reintervention than CABG (15, 16); (3) The inclusion criteria are limited. Only simple anatomy lesions may be appropriated for PCI.
CABG	(1) A lower rate of reintervention than PCI; (2) Suitable for complex lesions, such as giant aneurysms with multiple, ostial, or diffuse stenosis lesions	(1) Being more invasive with a longer recovery time; (2) CABG operation in children is technically demanding. Patient should be referred to an experienced tertiary referral center.

PCI, percutaneous coronary intervention; CABG, coronary artery bypass graft.

## Conclusion

Current advances in coronary artery intervention provide a broader spectrum of nonsurgical therapeutic options for young children with severe KD-related cardiac sequelae. Percutaneous recanalization of the coronary CTO followed by drug-coated ballooning is effective for pediatric patients with KD. More clinical experience and long-term follow-up are needed to assess the safety and efficacy of drug-coated balloons in this patient population.

## Data Availability

The original contributions presented in the study are included in the article/Supplementary Material, further inquiries can be directed to the corresponding author/s.
